# Genetic Variations in the Transforming Growth Factor Beta Pathway as Predictors of Bladder Cancer Risk

**DOI:** 10.1371/journal.pone.0051758

**Published:** 2012-12-12

**Authors:** Hua Wei, Ashish M. Kamat, Saad Aldousari, Yuanqing Ye, Maosheng Huang, Colin P. Dinney, Xifeng Wu

**Affiliations:** 1 Departments of Epidemiology, The University of Texas MD Anderson Cancer Center, Houston, Texas, United States of America; 2 Department of Urology, The University of Texas MD Anderson Cancer Center, Houston, Texas, United States of America; College of Pharmacy, University of Florida, United States of America

## Abstract

Bladder cancer is the fifth most common cancer in the United States, and identifying genetic markers that may predict susceptibility in high-risk population is always needed. The purpose of our study is to determine whether genetic variations in the transforming growth factor-beta (TGF-β) pathway are associated with bladder cancer risk. We identified 356 single-nucleotide polymorphisms (SNPs) in 37 key genes from this pathway and evaluated their association with cancer risk in 801 cases and 801 controls. Forty-one SNPs were significantly associated with cancer risk, and after adjusting for multiple comparisons, 9 remained significant (*Q*-value ≤0.1). Haplotype analysis further revealed three haplotypes within *VEGFC* and two haplotypes in *EGFR* were significantly associated with increased bladder cancer risk compared to the most common haplotype. Classification and regression tree analysis further revealed potential high-order gene-gene interactions, with *VEGFC:* rs3775194 being the initial split, which suggests that this variant is responsible for the most variation in risk. Individuals carrying the common genotype for *VEGFC*: rs3775194 and *EGFR*: rs7799627 and the variant genotype for *VEGFR*: rs4557213 had a 4.22-fold increase in risk, a much larger effect magnitude than that conferred by common genotype for *VEGFR*: rs4557213. Our study provides the first epidemiological evidence supporting a connection between TGF-β pathway variants and bladder cancer risk.

## Introduction

Bladder cancer commonly affects the elderly and men, with an estimated 73,510 new cases and 14,880 deaths in the United States in 2012 [1]. Major risk factors for bladder cancer include male gender, older age, tobacco smoking, and occupational exposure to aromatic amines [2]. It is increasingly recognized that genetic susceptibility may contribute to bladder cancer carcinogenesis [3]. Therefore, identifying individuals susceptible to cancer with the aid of genetic markers can reduce health care costs, increase the cost-benefit of screening and surveillance, and improve the treatment and survival of cancer patients.

The transforming growth factor-beta (TGF-β) pathway has been established playing important roles in different cancer types and implicated in the tumorigenesis of bladder transitional cell carcinoma. Many studies have indicated that TGF-β signaling contributes to epithelial-mesenchymal transition, angiogenesis, migration, and metastases in many types of malignant tumors [4] [5], [6]. In normal cells, TGF-β regulates cell growth, differentiation, matrix production, and apoptosis [7]. TGF-β induced apoptosis is frequently mediated by the smad-dependent pathway but may also occur through both p53-dependent and p53-independent mechanisms [8], [9], and involves caspase activation, upregulation of proapoptotic factors (i.e., Bax), and/or downregulation of antiapoptotic factors (i.e., Bcl-2 and Bcl-xL) [10]–[12]. These factors are all integral parts of the human immune system. The TGF-β receptor 1 variant rs11466445 (TGFBR1*6A) has been associated with an increased risk of breast and ovarian cancers but not colorectal or bladder cancer [13]–[16]. However, another study on the effects of 7 different genetic variants in two key members of the TGF- β pathway (*TGFB1* and *TGFBR1*) and the clinical outcome of muscle invasive bladder cancer indicated that *TGFBR1:* rs868, located in the 3′-untranslated region, was significantly associated with disease-specific mortality [17]. It is also reported that genetic variants in *RUNX3*, a tumor suppressor of the TGF- β pathway, may modulate bladder cancer risk [18].

Since the TGF-β pathway plays an essential role in cellular processes, we hypothesized that polymorphisms of TGF-β pathway genes may modulate the risk of bladder cancer. To test this hypothesis, we conducted a large case-control study to evaluate the effects of single-nucleotide polymorphisms (SNPs) in key genes from this pathway. To our knowledge, this is the first study to explore the association between a comprehensive panel of polymorphisms in the TGF-β pathway genes and bladder cancer risk and to identify subgroups that would be more likely to have higher cancer risk.

## Materials and Methods

### Ethics Statement

Written informed consents were obtained from all patients prior to enrollment in this study. The study was approved by the Institutional Review Boards at MD Anderson Cancer Center, Baylor College of Medicine, and Kelsey-Seybold Clinic.

### Study Population and Data Collection

This case-control study started patient recruitment in 1999 and is currently ongoing. Bladder cancer patients were recruited from the University of Texas MD Anderson Cancer Center and Baylor College of Medicine. The cases were patients with newly diagnosed, histologically confirmed bladder cancer. None of the patients had undergone chemotherapy or radiotherapy prior to study enrollment. There were no restrictions for age, gender, or disease stage at recruitment. The control subjects were healthy individuals without prior history of cancer (except nonmelanoma skin cancer). They were recruited from the Kelsey-Seybold clinic, the largest private multispecialty group practice in the Houston metropolitan area. Cases and controls were matched in terms of age (±5 years), sex and ethnicity. All study participants provided signed informed consent and completed a 45-minute in-person interview by trained MD Anderson staff interviewers. After each interview, a 40 ml peripheral blood sample was drawn into coded and heparinized tubes for subsequent DNA isolation and analysis. Individuals who had smoked less than 100 cigarettes in their lifetime were defined as never smokers, individuals who had smoked more than 100 cigarettes but had quit more than 1 year prior to diagnosis or interview were defined as former smokers, and individuals who were currently smoking or had stopped less than 1 year prior were defined as current smokers. In this study, former and current smokers were defined as ever smokers. Since more than 90% of our recruited cases were pure transitional cell carcinoma, we only included this histology in the study. Since more than 90% of our study population was self-reported non-Hispanic Caucasians based on the questionnaire date, we restricted the analysis to Caucasians to limit the confounding effect from population stratification while retaining most of the statistical power.

### SNP Selection and Genotyping

A panel of 356 SNPs in 37 genes (**Table S1**) was selected on the basis of the following criteria. Briefly, we utilized Ingenuity System Pathway Analysis software (http://www.ingenuity.com) and National Center for Biotechnology Information (NCBI) PubMed (http://www.ncbi.nlm.nih.gov) to identify a list of TGF-β pathway-related genes. For each gene, we selected tagging SNPs by the binning algorithm of LDSelect software (http://droog.gs.washington.edu/ldSelect.pl, version 1.0) (r^2^<0.8, MAF >0.05) within 10 kb upstream of the transcriptional start site or 10 kb downstream of the transcriptional stop site. SNP frequency and LD data were based on the International HapMap Project database, release 22, human genome build 36. We also included potentially functional SNPs with minor allele frequency (MAF) greater than 0.01 in the coding (synonymous and non-synonymous SNPs) and regulatory regions (promoter, splicing site, 5′-untranslated region, and 3′-untranslated region). The genotyping was performed using Illumina’s iSelect custom SNP array (Illumina, San Diego, CA). Genotypes were analyzed and exported using the Illumina BeadStudio software. Any SNPs with a call rate <95% was excluded from further analysis.

### Statistical Analysis

Laboratory and epidemiological data were merged and most analyses were performed using STATA 10.0 (Stata Corporation, College Station, TX) and HelixTree (Golden Helix, Bozeman, MT) software. Distributions of characteristics in cases and controls were evaluated by the χ^2^ test (for categorical variables) or Student’s t-test (for continuous variables). For each SNP, we tested Hardy-Weinberg equilibrium using the goodness-of-fit χ^2^ test to compare the observed with the expected frequency of genotypes in control subjects. The effects of genotypes of SNPs on bladder cancer risk were estimated as odds ratios and 95% confidence intervals (95% CI) using multivariate unconditional logistic regression under the dominant, recessive, and additive models of inheritance adjusted for age, gender, and smoking status, where appropriate. The best-fitting model was the one with the smallest *P* value among the three models. A *Q*-value was calculated to account for multiple comparisons. The *Q*-value measured the proportion of false positive incurred (false discovery rate) when a SNP shown significant. Bootstrap resampling was performed 100 times to internally validate the results [19]. Analysis of the combined effects of unfavorable genotypes involves a sum of all risk genotypes from those SNPs showing statistical significance in the main analysis (*P*<0.05) with equal contribution from each variant. Haplotype analysis was performed using the maximization algorithm implemented in the HelixTree software. Higher-order gene–gene interactions were evaluated using classification and regression tree (CART) analysis implemented in the HelixTree software. All statistical analyses were two-sided.

## Results

### Characteristics of the Study Population

As shown in **Table 1**, 801 Caucasian cases and 801 Caucasian controls were enrolled in this study. There were no significant differences in bladder cancer risk due to sex (*P* = 1) or age (*P* = 0.051). As predicted, cases reported a higher No. of Cigarettes/Day than controls (25.6 versus 22.5, *P*<0.001). Among smokers, cases reported higher pack-years of smoking than controls (43.0 versus 29.9, *P*<0.001). There was a significant difference between cases and controls by smoking status (*P*<0.001) with a higher percentage of current smokers in cases and higher percentage of never smokers in controls (*P*<0.001).

**Table 1:** Demographic and clinical variables for 801 cases and 801 controls.

Variables	Casesn = 801	Controlsn = 801	*P* * value
Sex (%)			
Male	638 (79.7)	638 (79.7)	
Female	163 (20.4)	163 (20.4)	1.0
Smoking Status (%)			
Never	212 (26.5)	355 (44.3)	
Former	403 (50.3)	379 (47.3)	
Current	186 (23.2)	67 (8.4)	<0.001
Smoked (%)			
Never	212 (26.5)	355 (44.3)	
Ever	589 (73.5)	446 (55.7)	<0.001
Mean Age, years (SD)	64.7 (11.1)	63.8 (10.9)	0.051
No. of Cigarettes/Day (SD)	25.6 (14.1)	22.5 (15.2)	<0.001
Smoking Pack-Years (SD)	43.0 (30.7)	29.9 (27.9)	<0.001

*P* values <0.05 were statistically significant.

* *P* values were derived from the χ^2^ test for the categorical variables gender, and smoking. Student’s *t*-test was used for the continuous variables age, no. of cigarettes, and pack-years.

### Individual SNPs and Overall Survival

The association of all significant SNPs with risk was summarized in **Table 2** (*P*<0.05). Among the 356 SNPs examined, 41 SNPs were significantly associated with cancer risk (*P*<0.05), and after adjusting for multiple comparisons, 9 remained significant, with a *Q*-value ≤0.1. These SNPs were located in three genes in the TGF- β pathway, including *VEGFC*, epidermal growth factor receptor gene (*EGFR*), and *SMAD3*.

**Table 2:** Association between TGF-β pathway genetic polymorphisms and bladder cancer risk.

				Cases/Controls						No. Times in bootstrap sample‡ *P*<0.05
SNP	Gene	Genotype	Best Model#	ww	wv+vv	vv	OR (95%CI)†	*P*	*Q*	
rs1485762*	*VEGFC*	G/A	ADD	363/422	351/316	87/67	1.42(1.19–1.69)	8.12×10^−5^	0.01	100
rs6593205*	*EGFR*	G/A	ADD	299/257	376/403	126/141	0.76(0.65–0.90)	1.17×10^−3^	0.09	95
rs6828869*	*VEGFC*	C/G	ADD	200/264	396/395	177/134	1.30(1.10–1.54)	1.91×10^−3^	0.1	100
rs12324036*	*SMAD3*	C/T	DOM	255/217	361/408	185/176	0.69(0.54–0.88)	2.6×10^−3^	0.1	100
rs3775194*	*VEGFC*	G/C	ADD	254/313	409/362	136/124	1.27(1.08–1.49)	3.28×10^−3^	0.1	98
rs11238349*	*EGFR*	G/A	ADD	396/441	326/296	79/63	1.28(1.08–1.51)	4.28×10^−3^	0.1	97
rs4557213*	*VEGFC*	A/G	DOM	631/670	163/124	7/7	1.50(1.13–1.99)	4.69×10^−3^	0.1	100
rs7799627*	*EGFR*	A/G	REC	392/368	344/340	65/93	0.59(0.41–0.86)	5.08×10^−3^	0.1	100
rs17697515*	*VEGFC*	G/A	DOM	670/703	123/93	8/5	1.57(1.14–2.17)	5.82×10^−3^	0.1	86
rs6969537	*EGFR*	G/A	DOM	600/562	191/223	10/16	0.72(0.56–0.92)	8.68×10^−3^	–	
rs11131764	*VEGFC*	A/G	DOM	639/673	156/122	6/6	1.45(1.09–1.93)	0.01	–	
rs9692301	*EGFR*	A/G	REC	393/394	316/342	92/64	1.60(1.11–2.31)	0.01	–	
rs1042265	*BAX*	C/T	DOM	641/686	153/109	7/6	1.47(1.09–2.00)	0.01	–	
rs2330951	*EGFR*	A/C	ADD	463/435	300/311	38/55	0.79(0.65–0.95)	0.01	–	
rs1792689	*SMAD2*	C/T	DOM	616/579	175/199	10/21	0.72(0.55–0.93)	0.01	–	
rs7176870	*SMAD3*	A/G	DOM	254/230	368/398	178/172	0.74(0.57–0.94)	0.01	–	
rs13222385	*EGFR*	A/G	ADD	306/338	373/370	122/93	1.22(1.04–1.43)	0.02	–	
rs2229995	*APC*	G/A	DOM	777/761	24/40	0/0	0.49(0.27–0.88)	0.02	–	
rs845558	*EGFR*	G/A	REC	258/284	381/382	162/135	1.44(1.07–1.94)	0.02	–	
rs718768	*EGF*	A/G	DOM	368/412	352/328	81/61	1.31(1.05–1.63)	0.02	–	
rs12907997	*SMAD3*	C/T	REC	217/219	376/403	208/179	1.37(1.06–1.79)	0.02	–	
rs4947972	*EGFR*	G/C	ADD	417/451	313/292	71/58	1.23(1.03–1.46)	0.02	–	
rs7801956	*EGFR*	G/A	DOM	655/684	142/113	4/4	1.42(1.05–1.91)	0.02	–	
rs1549854	*MAP2K1*	A/C	DOM	202/228	402/378	197/195	1.32(1.03–1.70)	0.03	–	
rs3743343	*SMAD3*	A/G	REC	422/439	321/327	58/35	1.72(1.04–2.85)	0.03	–	
rs763317	*EGFR*	G/A	DOM	205/237	409/400	186/162	1.32(1.02–1.69)	0.03	–	
rs7181936	*MAP2K1*	G/T	DOM	335/370	381/344	87/85	1.28(1.02–1.59)	0.03	–	
rs2110290	*EGFR*	A/G	ADD	375/393	338/331	85/74	1.20(1.01–1.43)	0.03	–	
rs2337146	*SMAD7*	C/T	DOM	762/738	37/63	2/0	0.62(0.40–0.97)	0.04	–	
rs11152377	*BCL2*	A/G	REC	259/265	408/371	134/165	0.73(0.55–0.98)	0.04	–	
rs1050171	*EGFR*	A/G	ADD	299/264	389/401	113/134	0.84(0.72–0.99)	0.04	–	
rs10488141	*EGFR*	A/T	REC	509/498	266/260	26/43	0.56(0.32–0.98)	0.04	–	
rs7226979	*BCL2*	C/T	REC	219/234	386/403	196/164	1.33(1.01–1.74)	0.04	–	
rs17697359	*VEGFC*	A/G	DOM	633/659	155/133	13/9	1.34(1.01–1.76)	0.04	–	
rs42427	*APC*	A/G	REC	316/327	373/385	112/89	1.40(1.01–1.93)	0.04	–	
rs11568993	*EGF*	C/T	DOM	659/684	142/115	0/2	1.35(1.01–1.81)	0.04	–	
rs759160	*EGFR*	A/G	REC	477/435	277/302	47/63	0.63(0.40–0.99)	0.04	–	
rs845561	*EGFR*	A/G	REC	472/458	289/285	40/57	0.61(0.37–1.00)	0.05	–	
rs984654	*EGFR*	A/G	ADD	427/458	312/289	62/53	1.19(1.00–1.42)	0.05	–	
rs7964492	*INHBC*	A/C	REC	494/480	273/270	31/47	0.59(0.35–1.01)	0.05	–	

* SNPs that remained significant after controlling for multiple comparisons by *q*-value, with false discovery rate <10%.

# Best model: the model with the smallest *P* value; ADD: additive model, DOM: dominant model, RES: recessive model.

† Adjusted by age, sex, and smoking status.

‡ Non-significant SNPs (by *Q*-value) were not tested using the bootstrapping method.

To internally validate these results, we next performed random bootstrap sampling of the significant SNPs for 100 iterations and listed the number of times that the *P* value was <0.05. Eight of these nine top SNPs had highly consistent results, with bootstrap *P* values <0.05 for greater than 90% of the samplings (**Table 2**).

### Cumulative Effects of Multiple Unfavorable Genotypes on Cancer Risk

Because abnormal TGF-β signaling can result in the activation of multiple downstream genes and 9 SNPs reached statistical significance after multiple comparisons in the main effect analysis, we used combined analysis to determine whether multiple unfavorable genotypes in the TGF-β pathway have an additive effect on bladder cancer risk. There was a significant dose–response trend of increased risk of bladder cancer with increasing number of unfavorable genotypes. Compared with the reference group consisting of subjects with 0∼2 unfavorable genotypes, the groups with 3 or 4 unfavorable genotypes had a significantly elevated risks with the ORs of 1.73 (95% CI, 1.28–2.33, *P*<0.001) or 2.15 (95% CI, 1.59–2.91, *P*<0.001), respectively, whereas the high-risk group with 5∼9 unfavorable genotypes had a significantly elevated risks with the OR of 2.57 (95% CI, 1.92–3.43, *P*<0.001) (*P*_trend_ = 1.07×10^−10^) (**Table 3**).

**Table 3:** Cumulative effect of unfavorable genotypes on bladder cancer risk.

#No. of genotypes	Case,n (%)	Control,n (%)	OR (95% CI)*	*P* * value
0∼2	142 (36.79)	244 (63.21)	1 (Ref.)	
3	185 (48.94)	193 (51.06)	1.73 (1.28–2.33)	3.42×10^−4^
4	201 (54.77)	166 (45.23)	2.15 (1.59–2.91)	6.58×10^−7^
5∼9	263 (58.44)	187 (41.56)	2.57 (1.92–3.43)	1.57×10^−10^
*P* for trend				1.07×10^−10^

* Adjusted for age, sex, smoking status.

# Unfavorable genotypes: rs1485762(AA+GA), rs3775194(CC+CG), rs11238349(AA+AG), rs4557213(GG+AG), rs17697515(AA+GA), rs6828869(GG), rs7799627(AA+AG), rs12324036(GG) and rs6593205(GG).

### Haplotype Analysis of VEGFC and EGFR SNPs

As multiple SNPs in the *VEGFC* and *EGFR* genes showed significant associations, we next performed haplotype analysis for 8 top SNPs in these genes in our study population. We observed three haplotypes significantly associated with bladder cancer risk (**Table 4**). Haplotype H7 was composed of SNPs rs1485762-rs6828869-rs17697515-rs3775194-rs4557213, and subjects carrying rs1485762 wildtype, rs6828869 variant, rs17697515 wildtype, rs3775194 variant, and rs4557213 wildtype alleles showed a significant 78% increase in risk (OR, 1.78; 95%CI,1.13–2.82; *P = *1.4×10^−2^) compared to those carrying haplotype H0. Haplotypes H11 andH15 also reached significant associations with bladder cancer risk with OR of 1.63 (95%CI, 1.09–2.43; *P* = 1.6×10^−2^) and OR of 1.78 (95%CI, 1.23–2.57; *P* = 2.0×10^−3^) respectively (**Table 4**). Since three SNPs in the *EGFR* gene showed significant associations after multiple comparisons (*Q* ≤0.1, **Table 2**), we also performed haplotype analysis for these three SNPs in *EGFR* in total population and identified two significant haplotypes (*P*<0.05) (**Table 5**). Haplotype H3 was composed of SNPs rs11238349- rs7799627- rs6593205, and subjects carrying rs11238349 wildtype, rs7799627 variant, and rs6593205 variant alleles showed a significant 25% decrease in risk (OR, 0.75; 95%CI, 0.56–1.00; *P* = 0.05) compared to haplotype H0. Haplotype H4 also reached a significant association with bladder cancer risk with an OR of 1.41 (95%CI, 1.09–1.83; *P* = 1.0×10^−2^) (**Table 5**).

**Table 4:** Association between *VEGFC* haplotypes and bladder cancer risk.

*VEGFC*	Case,(n %)	Control,(n %)	AdjustedOR(95%CI)*	*P* value
H0: GCGGA	416(36.62)	518(43.09)	1(Ref)	
H1: GCGGG	2(0.18)	6(0.50)	0.52(0.10–2.66)	0.43
H2: GCGCA	196(17.25)	215(17.89)	1.10(0.86–1.40)	0.45
H3: GCGCG	25(2.20)	21(1.75)	1.42(0.77–2.62)	0.26
H4: GCAGA	1(0.09)	0	N/A	
H5: GCACG	1(0.09)	1(0.08)	1.17(0.07–18.74)	0.91
H6: GGGGA	88(7.75)	99(8.24)	1.06(0.77–1.47)	0.71
**H7: GGGCA**	**49(4.31)**	**37(3.08)**	**1.78(1.13–2.82)**	**1.4**×**10** ^−**2**^
H8: GGGCG	2(0.18)	0	N/A	
H9: ACGGA	5(0.44)	1(0.08)	7.95(0.90–69.93)	0.06
H10: AGGGA	166(14.61)	166(13.81)	1.26(0.97–1.63)	0.08
**H11: AGGCA**	**67(5.90)**	**52(4.33)**	**1.63(1.09–2.43)**	**1.6**×**10** ^−**2**^
H12: AGGCG	7(0.62)	8(0.67)	1.12(0.39–3.16)	0.84
H13: AGAGA	17(1.50)	12(1.00)	1.81(0.83–3.94)	0.14
H14: AGACA	11(0.97)	5(0.42)	2.57(0.85–7.76)	0.09
**H15: AGACG**	**83(7.31)**	**61(5.07)**	**1.78(1.23–2.57)**	**2.0**×**10** ^−**3**^

Note: rs1485762-rs6828869-rs17697515-rs3775194-rs4557213.

* Adjusted for age, sex, smoking status.

**Table 5:** Association between *EGFR* haplotypes and bladder cancer risk.

*EGFR*	Case, (n %)	Control, (n %)	Adjusted OR(95%CI)*	P value
H0: GAG	318(29.12)	305(28.50)	1(Ref)	
H1: GAA	141(12.91)	154(14.39)	0.83(0.62–1.11)	0.20
H2: GGG	162(14.84)	180(16.82)	0.87(0.66–1.14)	0.31
**H3: GGA**	**136(12.45)**	**167(15.61)**	**0.75(0.56–1.00)**	**0.05**
**H4: AAG**	**239(21.89)**	**166(15.51)**	**1.41(1.09–1.83)**	**0.01**
H5: AAA	95(8.70)	97(9.07)	0.95(0.68–1.33)	0.76
H6: AGA	1(0.09)	1(0.09)	1.23(0.07–20.26)	0.89

Note: rs11238349-rs7799627-rs6593205.

* Adjusted for age, sex, smoking status.

### Higher-order Gene-gene Interactions

**Figure 1:**
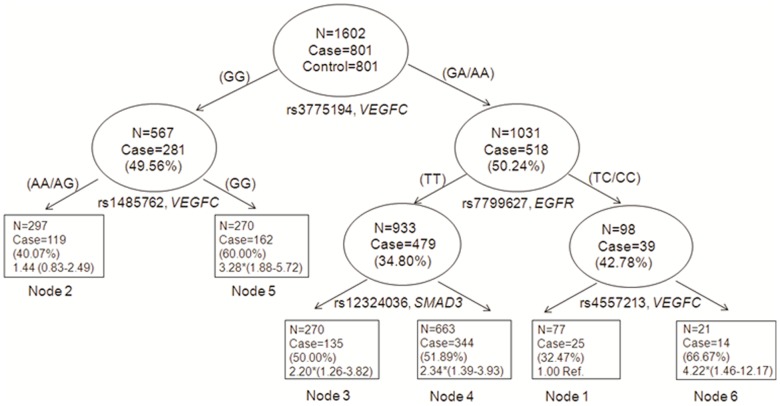
Higher-order gene-gene interactions and bladder cancer risk. Note: Each node denotes number and percentage of cases and OR with 95% confidence interval in parenthesis. *Significant at *P*<0.05.

We next explored higher-order gene-gene interactions to determine whether or not complex interactions among these significant SNPs could further modulate bladder cancer risk. The final tree structure identified several potential interactions among the top nine SNPs (**Figure 1**). *VEGFC*: rs3775194 was identified as the initial split, which suggests its important role in gene-gene interaction and the potential to predict cancer risk. The final tree structure also identified four terminal nodes with significantly higher risk than the low-risk genetic profile of node 1 (**Figure 1**). In particular, node 5 had a significantly elevated risk with the OR of 3.28 (95% CI, 1.88–5.72), while node 6 had an even higher significantly elevated risk with the OR of 4.22 (95% CI, 1.46–12.17).

## Discussion

Bladder cancer remains to be a challenging disease due to the high rate of recurrence and the accompanied high medical costs. Using genetic markers for determining risk may help to identify high risk population for early screening, diagnosis, and therapy, which may improve clinical outcome. This is the first study to explore the association between a comprehensive panel of polymorphisms of TGF-β pathway genes and bladder cancer risk and to identify subgroups that would more likely have higher cancer risk. We identified and evaluated 356 SNPs in 37 key genes from the TGF-β pathway for their associations with bladder cancer risk. Our results identified 41 SNPs significantly associated with bladder cancer susceptibility, and nine of them remained significant after adjustment for multiple comparisons (*Q* ≤0.1). In particular, SNPs in *VEGFC* showed the most significant associations in single SNP and haplotype analyses. CART analysis further revealed potential high-order gene-gene interactions and categorized subjects into different risk groups according to their specific polymorphic signatures. Our study provides the first epidemiological evidence supporting a connection between a comprehensive TGF-β pathway SNPs and bladder cancer risk.

Five of the top nine significant SNPs were in *VEGFC*, which is known to have important functions contributing to bladder cancer risk (Table 2). VEGFC is a member of the platelet-derived growth factor/vascular endothelial growth factor (PDGF/VEGF) family. This gene functions in angiogenesis, lymphangiogenesis, endothelial cell growth and survival, affects the permeability of blood vessels, and also facilitates nodal metastasis. Several studies have correlated elevated VEGF expression with disease recurrence or progression, often as an independent predictor on multivariate analysis. There is a positive correlation between *VEGF-C* expression and lymphatic invasion in patients with breast, gastric, and cervical cancer [20]–[22]. VEGF-C expression was found in the cytoplasm of transitional carcinoma cells and was associated with lymph node metastasis in bladder cancer [23]–[24] and also has been found to correlate with clinical parameters like tumor size, pathological T stage, pathological grade, lymphatic-venous involvement, and pelvic lymph node metastasis in bladder cancer patients [23]. Haplotype analysis further identified three significant haplotypes of *VEGFC*, which suggests that haplotype-based analysis may be more informative than single SNP analysis and resequencing DNA samples carrying the high-risk and low-risk haplotypes may be able to improve risk assessment. The five most significant (*Q* <0.1) *VEGFC* SNPs we identified are all located in the intron region, which may contribute to alterations in gene expression or splicing. Alternatively, it is also possible that these SNPs are linked to other causal variants in *VEGFC*.

Three of the nine most significant SNPs were in *EGFR* (**Table 2**). EGFR is a tyrosine kinase transmembrane receptor in the ErB family of receptors expressed on the surface of epithelial cells [25]. EGFR regulates important processes in carcinogenesis, including cell survival, tumor invasion, and angiogenesis and is involved in many malignancies, including bladder cancer [26]. EGFR overexpression is frequently observed in tumors and pre-cancerous lesions and induces tumor formation in animal studies. EGFR expression in bladder cancer independently predicts disease progression and mortality, and both VEGF and EGFR are emerging as important targets for the treatment of metastatic bladder cancer [25]. It is possible that these EGFR SNPs may affect gene transcription, thus altering protein level, or they may be linked to other causal variants in *EGFR.* In addition, we also identified *SMAD3*: rs12324036 significantly associated with cancer risk. It is well known that TGF-β/Activin/Nodal signaling is transduced by SMAD2 and SMAD3, and increased TGF-β1 level can revert a malignant phenotype to a less aggressive phenotype in rat bladder carcinoma cell line lacking TGF-β1 [27]. Our previous study also identified *SMAD3*: rs11632964 being significantly associated with lung cancer overall survival [28], which also highlights the important role of SMAD3 in cancer. It is possible that the variant allele of *SMAD3:* rs12324036 may affect gene transcription thus altering protein level. Alternatively, it may be linked to other causal variants in *SMAD3*. Overall, our study highlights the association of *EGFR VEGFC*, and *SMAD3* polymorphisms with bladder cancer risk.

We next applied a pathway-based approach to comprehensively evaluate the effect of the nine significant SNPs (*Q* <0.1) on the risk of bladder cancer. We identified a gene-dosage effect for the nine SNPs that were significant after multiple comparison adjustment. Those with 5∼9 risk genotypes had the highest risk of bladder cancer, suggesting additional variations within this key pathway was detrimental and had a larger effect than any single variant. Furthermore, the magnitude of each individual SNP was modest, but the risk for individuals with five to nine of these risk genotypes was more than doubled. This also emphasizes the importance of including multiple SNPs within a shared pathway for examining joint effects in the risk assessment.

Within the framework of a pathway, we hypothesized that gene-gene interactions would further modulate the risk of bladder cancer. Potential gene-gene interactions among three variants were observed, with *VEGFC:* rs3775194 being the initial split in our CART analysis, suggesting its importance in determining the most variation in risk. Individuals carrying the common genotype for *VEGFC*: rs3775194 and *EGFR*: rs7799627 and the variant genotype for *VEGFR*: rs4557213 had a 4.22-fold increase in risk, a much larger effect magnitude than that conferred by common genotype for *VEGFR*: rs4557213.

In summary, we have performed a pathway-based analysis of TGF-β pathway genes and bladder cancer risk. These data provide important genetic information for predicting individuals at risk for bladder cancer and identifying tumors at an early, curable stage. In addition, our relatively comprehensive query of TGF-β pathway polymorphisms and our large population with detailed risk information provide substantial evidence for the involvement of SNPs as predictors or modulators of bladder cancer risk. However, there are some limitations in our study. For example, further fine mapping and functional assays are necessary to reveal potential molecular mechanisms of these SNPs or other linked causal polymorphisms. Additionally, only Caucasians were included in this study. It would be interesting to exam these SNPs in minority populations. Finally, although our data are largely internally validated, future replication studies in independent populations are needed to validate some of the results.

## Supporting Information

Table S1 Selected genes of the TGF-β pathway for this study.
(DOC)
