# Supplementary material for: Genetic Variations in the Transforming Growth Factor Beta Pathway as Predictors of Bladder Cancer Risk
Source: PLoS One. 2012 Dec 12;7(12):e51758. doi: 10.1371/journal.pone.0051758 (PMC3520916; doi:10.1371/journal.pone.0051758)
Supplement: Table S1 — Selected genes of the TGF-β pathway for this study. (DOC) [file pone.0051758.s001.doc]

**Supplemental Information**

**Table S1.** Thirty seven key genes from TGF-β pathway were evaluated for association with bladder cancer risk in 801 cases and 801 controls.

| Gene name |
| --- |
| ACVR1 |
| ACVR1B |
| APC |
| BAX |
| BCL2 |
| BMP1 |
| BMP15 |
| BMP2 |
| BMP3 |
| BMP4 |
| BMP7 |
| EGF |
| EGFR |
| INHA |
| INHBA |
| INHBB |
| INHBC |
| MAP2K1 |
| MAPK3 |
| PITX2 |
| SERPINE1 |
| SMAD1 |
| SMAD2 |
| SMAD3 |
| SMAD4 |
| SMAD5 |
| SMAD6 |
| SMAD7 |
| TGFB1 |
| TGFB2 |
| TGFB3 |
| TGFBR1 |
| TGFBR2 |
| VEGFA |
| VEGFB |
| VEGFC |
| ZFYVE9 |
